# Application of antibiotic-loaded bone cement in treating lower limb soft tissue infection

**DOI:** 10.12669/pjms.41.3.9872

**Published:** 2025-03

**Authors:** Zhigang Lang, Xu Zhang, Yuxiang Liang

**Affiliations:** 1 Zhigang Lang Department of Orthopedics, Sichuan Orthopedic Hospital, Chengdu 610041, Sichuan, China; 2 Xu Zhang Department of Orthopedics, Sichuan Orthopedic Hospital, Chengdu 610041, Sichuan, China; 3 Yuxiang Liang Department of Orthopedics, Sichuan Orthopedic Hospital, Chengdu 610041, Sichuan, China

**Keywords:** Antibiotic-loaded bone cement, Lower limb, Soft tissue infection

## Abstract

**Objective:**

To explore the clinical efficacy of antibiotic-loaded bone cement in the treatment of lower limb soft tissue infection.

**Methods:**

A retrospective analysis was carried out on 40 patients with lower limb soft tissue infection who were admitted to Sichuan Orthopedic Hospital from January 1, 2022 to December 1, 2023. The enrolled patients were divided into two groups according to different therapies, including the bone cement treatment (the study group) group and the conventional treatment group (the control group), with 20 cases in each group. Further comparison was made on the number of operations, wound healing time, Grade-A wound healing rate, recurrence rate, and recovery rate of inflammatory indicators between the two groups.

**Results:**

Patients in both groups were followed up, and there was no statistically significant difference in the number of operations between the two groups (P>0.05). The study group had shorter wound healing time (P<0.05), higher Grade-A wound healing rate (P<0.05), lower recurrence rate (P<0.05), and higher normal rate of inflammatory indicators (P<0.05) than those in the control group.

**Conclusion:**

Antibiotic-loaded bone cement showed a clear effect on treating lower limb soft tissue infection, with good wound healing and low recurrence rate.

## INTRODUCTION

Generally, lower limb soft tissue infection is secondary to trauma, diabetes, and local skin ulcers, with a high incidence rate.^1^-^3^ Especially for the elderly and the diabetics with poor local blood circulation, there may be a high risk of developing local abscesses, necrosis and skin ulcers once infected, which is difficult to heal voluntarily. Limb infection commonly requires surgical debridement and drainage, supplemented by systemic antibiotic infusion.^4^,^5^

However, there may be residual cavities after soft tissue debridement, especially in deep soft tissues where the residual cavities are large and irregular, which can lead to poor drainage and thus poor infection control. Significantly, antibiotic-loaded bone cement has its unique advantages in the treatment of bone and joint infections, yet with relatively few research on the treatment of soft tissue infection. Therefore, the present study retrospectively analyzed the clinical data from 40 patients with lower limb soft tissue infection who were admitted to Sichuan Orthopedic Hospital from January 1, 2022 to December 1, 2023. This study compared the therapeutic effect of conventional debridement and drainage with antibiotic-loaded bone cement to fill in residual cavities after debridement. It is reported as follows.

## METHODS

A retrospective analysis was carried out on 40 patients with lower limb soft tissue infection who were admitted to Sichuan Orthopedic Hospital from January 1, 2022 to December 1, 2023 and divided into the study group (n=20) and the control group(n=20) according to different therapies.

### Ethical Approval:

The study was approved by the Institutional Ethics Committee of Sichuan Orthopedic Hospital (No.:2017-6-30-1; Date: June 30, 2017), and written informed consent was obtained from all participants.

### Inclusion criteria:

- Patients diagnosed clinically with lower limb soft tissue infection.
- Patients without bone infection.
- Patients without implants in the infected area.

### Exclusion criteria:

- Patients with simple skin infections and ulcers.
- Patients with diabetes and lower extremity arterial disease.
- Patients who were unable to receive primary suture of the incision.

The study included 40 patients, including 15 males and 25 females, the age ranged between 15~62 years (average of 28 years) and the duration ranged between 9-32 days. Among them, 17 cases had lesions in the thigh and 23 cases in the calf. There were 29 cases of post-traumatic infection, 6 cases of secondary ulcer infection, and 5 cases of other infections. In addition, there were 33 patients with concurrent wounds or sinus tract, and both groups of patients had abnormal inflammatory markers to varying degrees detected upon admission. There was no statistically significant difference in clinical data comparison between the two groups (*P>*0.05, Table-I).

**Table-I:** Comparison of clinical data between the two groups of patients.

Items	Site (thighs/calves)	Age (years)	Duration (days)	Concurrent sinus tract (rate)
Study group (n=20)	9/11	30.47±4.51	17.72±5.63	17 (85%)
Control group (n=20)	8/12	32.76±3.49	16.36±4.69	16 (80%)
*t/χ* ^2^	0.732	-0.628	1.209	0.676
*P*	0.365	0.531	0.340	0.478

### Before operation:

Both groups of patients completed preoperative routine examinations and detection of infection indicators such as routine blood test, erythrocyte sedimentation rate, and C-reactive protein, to exclude surgical contraindications and identify the range of lesions. Patients with concurrent wound formation were required to receive bacterial culture of secretions from the affected area and drug sensitivity test, combined with nutritional supplementation to provide favorable conditions for surgery.

### During operation:

Thorough debridement was performed in the affected lesion area, including within the bone defect lesion and within 1 cm of the edge. The operation was initiated by routine removal of infection, necrosis, and diseased tissue within the surgical area until fresh exudation was visible on the wound surface. Infected and necrotic tissues from three different parts were collected and sent for bacterial culture and drug sensitivity test to guide the use of antibiotics postoperatively. The surgical cavity was then rinsed with povidone iodine(iodine) and hydrogen peroxide (hydrogen peroxide).

After that, the surgical cavity was rinsed with a large amount of physiological saline (3-6 L) through low-pressure pulse rinse apparatus. After debridement, the control group was given direct placement and fixation of a vacuum-assisted drainage tube inside the incision, followed by primary suture of the incision and dressing with compression. While the study group applied a mixture of sensitive antibiotic powder and bone cement powder based on preoperative results of drug sensitivity test and bacterial culture. For patients with false negative culture result or the culture cannot be taken from patients, vancomycin and tobramycin powder should be mixed evenly in a ratio of 0.5:10 (i.e. containing 5% antibiotic bone cement)^6^, followed by the addition of bone cement monomer for thorough mixing. When the bone cement entered the dough stage, it was shaped according to the size and shape of the residual cavity (mostly in the form of flakes or blocks).

After shaping, it was filled in the residual cavity as much as possible to fill the soft tissue residual cavity after debridement. Finally, a vacuum-assisted drainage tube was placed inside the incision and fixed for drainage, followed by primary suture of the incision. Relaxation suture should be applied in case of high skin tension on the wound.

### After operation:

The two groups of patients underwent routine dressing on their incisions, with the healing and drainage volume of the incisions observed. When there was exudation from the incisions, secretions were collected for routine bacterial culture and drug sensitivity test. When the drainage flow rate was less than 15ml/24 hours, the drainage tube can be removed, and the suture was removed postoperatively after the healing of the incision. In the study group, another surgery was performed to remove the bone cement four weeks after the complete healing of the incision. The two groups of patients were provided with continued systemic infusion of antibiotics based on intraoperative bacterial culture results for two weeks, followed by oral administration for two weeks. The intraoperative results should be referred if the intraoperative bacterial culture results were inconsistent with those preoperatively. The follow-up work of all patients was completed by the same group of surgeons.

### Outcome measures:

This study observed and recorded the number of operations, incision healing time, healing grade, infection recurrence rate, and recovery of inflammatory indicators in the two groups of patients. The healing of the incision was divided into three grades based on the evaluation criteria of surgical incision healing:

1. Grade-A healing: Grade-A healing indicated excellent healing with no adverse reactions.
2. Grade-B healing: Grade-B healing referred to the presence of inflammatory reactions (e.g., redness, swelling, hematoma, fluid accumulation, etc.) at the healing site, but without suppuration, with primary healing achieved after active treatment.
3. Grade-C healing: Grade-C healing suggested purulent incision that required incision drainage to promote healing.

### Follow-up:

After discharge, patients were followed up continuously for three months (once a month) to evaluate local healing, routine blood test, erythrocyte sedimentation rate, and C-reactive protein levels.

### Statistical analysis:

Data analysis of this study employed SPSS 21.0 software. Comparison of measurement data and counting data used *t*-test and *χ*^2^ test, respectively. P<0.05 indicated the presence of a statistically significance difference.

## RESULTS

As for treatment efficacy, all 20 patients in the study group showed relatively satisfactory overall treatment effect, and 19 patients had Grade-A wound healing. The remaining one patient experienced postoperative incision exudation and no healing. Bacterial culture in this patient indicated Klebsiella pneumoniae, and antibiotic-loaded bone cement was re implanted after debridement. With prolonged duration of drainage tube placement, this case showed delayed healing of the incision for 21 days. No case of recurrence was found during the three months follow-up; additionally, one patient had an abnormal erythrocyte sedimentation rate, and all other patients revealed no abnormality in inflammatory markers.

Furthermore, in the control group, there were 13 cases of Grade-A wound healing, and the other seven cases had poor infection control, with unhealed incisions showing redness, swelling, and exudation. Among them, two cases were treated with partial suture removal and drainage, dressing change, adjustment of antibiotic infusion, and ultimately showed delayed healing. The additional five cases underwent re-operation, and achieved wound healing finally after adopting antibiotic-loaded bone cement implantation. The three months follow-up indicated that three patients had recurrent infections and elevated inflammatory markers four to six weeks after discharge, while two patients had their erythrocyte sedimentation rate not reduced to normal. The specific comparison is as follows:

There was no statistically significant difference in the number of operations between the two groups (P>0.05). The study group had shorter wound healing time (P<0.05), higher Grade-A wound healing rate (P<0.05), lower recurrence rate (P<0.05), and higher normal rate of inflammatory indicators (P<0.05) than those in the control group. Table-II.

**Table-II:** Comparison of therapeutic effects between the two groups of patients [n (%)].

Groups	Number of operation (times)	Healing time (days)	Grade-A healing rate	Recurrence rate	Normal rate of inflammatory indicators
Study group (n=20)	2.36±0.25	17.47±3.25	19 (95)	0 (0)	19 (95)
Control group (n=20)	2.87±0.54	29.25±4.18	13 (65)	3 (15)	15(75)
*t/χ* ^2^	1.575	1.367	2.489	4.751	5.293
*P*	0.476	0.013	0.021	0.032	0.036

**Fig.1:**
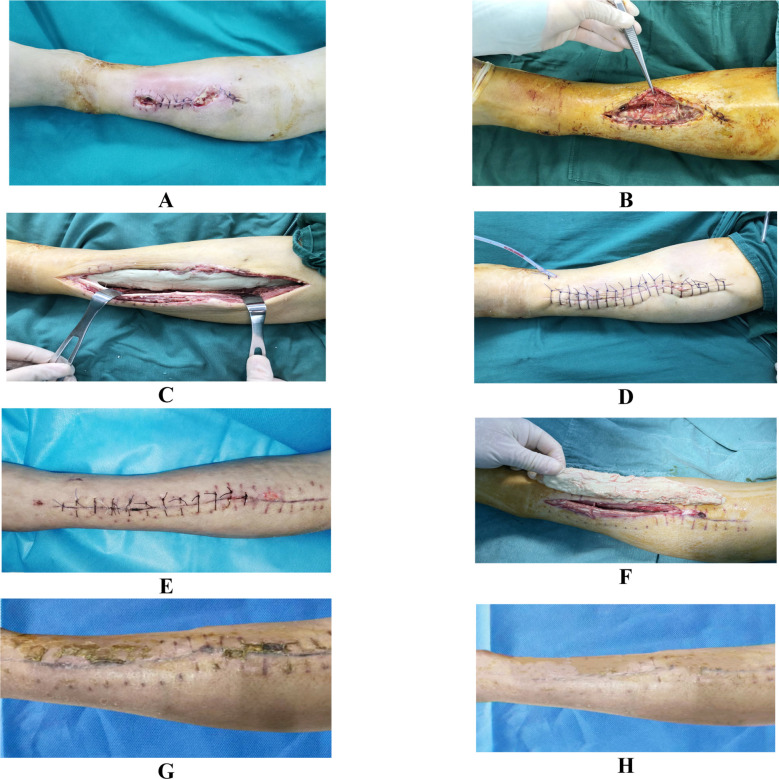
Preoperative and postoperative images of a typical patient. A: Preoperative appearance, with infected limb incision, rupture and pus discharge, B: Subcutaneous soft tissue infection and necrosis during intraoperative debridement, with a large amount of pus substance, C: Thorough debridement and filling of the residual soft tissue cavity with vancomycin-loaded bone cement, D: Primary suture of the surgical incision, with further placement of drainage tube, E: Wound healing and suture removal two weeks after operation, F: Removal of the implanted bone cement four weeks after incision healing and suture removal, G: Good healing of the incision and preparation for suture removal two weeks after the removal of bone cement, H: Surgical appearance during the follow-up at three months after operation.

A 46 years old female patient had debridement surgery for left calf injury caused by trauma for 15 days, with incision redness, swelling, and exudation for eight days, and re-debridement for four days. The patient showed no healing of incision. After admission, bacterial culture suggested methicillin-resistant Staphylococcus aureus, with sensitivity to vancomycin. Routine blood test results: white blood cell count of 12.5×10^9^/L, neutrophil percentage of 86.3%, C-reactive protein of 67.9mg/L, and erythrocyte sedimentation rate of 91.3mm/h. After admission, the patient underwent extensive debridement of the infected lesion. After debridement, 20g of 5% vancomycin-loaded bone cement was used to form a strip-shaped filling of the residual cavity. With primary suture after the operation, the wound was fully healed two weeks postoperatively. Another operation was performed to remove the bone cement 28 days after discharge, and the patient achieved satisfactory treatment results finally, as shown in Fig.1.

## DISCUSSION

In this study, there was no significant difference in the comparison of the number of operations between the study group and the control group. However, in terms of infection control, the study group exhibited a greater advantage in promoting incision healing and reducing recurrence. The majority of patients in the study group achieved Grade-A healing of the incision within the expected time after primary debridement and suture. The comparability of the number of operations with that of the control group may be related to the need for further surgery to remove bone cement in the later stage. However, in the control group, seven patients experienced non-healing wounds after primary debridement and drainage, and had to undergo long-term dressing change and reoperation for further treatment. Consequently, it resulted in prolonged healing of the incision and increased number of operations. While the study group had antibiotic-loaded bone cement implanted after debridement to effectively fill the residual cavity, which could prevent the formation of dead space. Moreover, the vacuum-assisted drainage tube placed in the incision directly drained the lesion to prevent local fluid and blood accumulation, which could effectively prevent infection recurrence. Thorough debridement constitutes the prerequisite for treating soft tissue infection.^7^,^8^ Residual cavities remain inevitably after debridement, leading to a higher risk of blood and fluid accumulation that provide favorable conditions for bacterial reproduction. Therefore, eliminating the residual cavity is crucial in the process of infection treatment. Indeed, dressing with compression at the side of incision can indirectly narrow the residual cavity.

However, some residual cavities are deep and large with irregular shape, the proposed method of dressing may not be uniform and sufficient, accompanied by blood and fluid accumulation still in the residual cavity. For instance, it is a challenge to apply dressing with compression for infection in the proximal thigh due to the special anatomical features. Meanwhile, patients with lesions close to the knee joint are prone to loosening and failure of dressing with compression due to joint movement. In addition, as reported previously, some researchers have suggested the use of vacuum-assisted closure drainage or direct coverage of the incision surface by dressing with vacuum-assisted drainage function to compress the residual cavity and drainage postoperatively. While there was no significant difference in the comparison of its therapeutic effect with that of conventional dressing.^9^-^11^

While antibiotic-loaded bone cement has dual effects of eliminating residual cavities and controlling infection locally. Specifically, antibiotic-loaded bone cement can obtain 10-100 times the effective concentration of antibiotics, which can deactivate local residual bacteria effectively.^12^-^15^ Moreover, its effective concentration can be continuously released for a period of >2 weeks, which can meet the treatment cycle for soft tissue infection.^16^ Simultaneously, bone cement can be a spacer that fully fills the residual cavity after debridement, ensuring the maximum effect of thorough debridement and preventing infection recurrence.

In 1970, Buchholz and Engelbrecht^17^, for the first time, proposed the concept of incorporating antibiotics into bone cement to prevent infection after joint replacement. Prior experiment has confirmed that the release of antibiotics on the surface of bone cement and around the implant can act as a barrier to bacterial growth.^18^ In recent decades, there has been extensive use of antibiotic-loaded bone cement in the treatment of orthopedic infections with significant therapeutic effects, However, it is less commonly used in treating soft tissue infections. Similarly, in our study, antibiotic-loaded bone cement was used to locally fill the residual cavity and release antibiotics to treat lower limb soft tissue infection. Treating infections with local antibiotics is a long-standing method, which requires the use of carriers frequently to exert anti-infective effect.^19^ Bone cement is a non-degradable carrier that can deliver various types of antibiotics to meet different anti-infection needs clinically.^20^

The bone cement used in this study did not contain antibiotics and needed to be added intraoperatively. For use, antibiotics that can be artificially added to bone cement shall have the following characteristics: 1. powder, highly soluble in water; 2. fairly heat-stable; 3. with no significant effect on the mechanical strength and chemical properties of bone cement; and 4. with excellent release rate from solidified bone cement.

In view of our experience in selecting antibiotics, the first- and second-generation cephalosporins, or vancomycin should be used for patients with Gram-positive bacteria based on drug sensitivity test; while the third- and fourth-generation cephalosporins, or tobramycin can be applied for patients with Gram-negative bacteria. In addition, for some patients with the failure of bacterial culture preoperatively, we may utilize two antibiotics, e.g., vancomycin combined with tobramycin, for Gram-positive bacteria and Gram-negative bacteria, respectively.

### Limitations:

However, due to the un-degradability of bone cement, a secondary operation is required for surgical removal around four weeks after complete healing of the incision, which increases the number of operations for this method. Besides, this study was performed based on a smaller sample size, which may produce bias in the results. Further larger-scale and multi-center comparative studies will be conducted in the future, with optimized experimental protocol, so as to obtain more accurate experimental results to guide clinical practice.

## CONCLUSIONS

In conclusion, we report the application of antibiotic-loaded bone cement in treating patients with lower limb infections, with good patient experience, effective infection control, relatively short incision healing time, and high Grade-A healing rate. It exhibits a more significant effect especially in patients with deep thigh infection combined with soft tissue defect.

### Authors’ Contributions:

**ZL:** Carried out the studies, participated in collecting data, and drafted the manuscript, and are responsible and accountable for the accuracy or integrity of the work.

**XZ** and **YL:** Performed the statistical analysis , participated in its design. Critical Review.

All authors have read and approved the final manuscript.
